# Circulating Polymorphonuclear Myeloid-Derived Suppressor Cells (PMN-MDSCs) Have a Biological Role in Patients with Primary Myelofibrosis

**DOI:** 10.3390/cancers16142556

**Published:** 2024-07-16

**Authors:** Rita Campanelli, Adriana Carolei, Paolo Catarsi, Carlotta Abbà, Emanuela Boveri, Marco Paulli, Raffaele Gentile, Monica Morosini, Riccardo Albertini, Stefania Mantovani, Margherita Massa, Giovanni Barosi, Vittorio Rosti

**Affiliations:** 1 Center for the Study of Myelofibrosis, Fondazione IRCCS Policlinico San Matteo, 27100 Pavia, Italy; a.carolei@smatteo.pv.it (A.C.);; 2 General Medicine 2-Center for Systemic Amyloidosis and High-Complexity Diseases, Fondazione IRCCS Policlinico San Matteo, 27100 Pavia, Italy; 3 Unit of Anatomic Pathology, Fondazione IRCCS Policlinico San Matteo, 27100 Pavia, Italy; 4 Department of Molecular Medicine, Unit of Anatomic Pathology, University of Pavia, 27100 Pavia, Italy; 5 Chemical and Clinics Laboratory, Fondazione IRCCS Policlinico San Matteo, 27100 Pavia, Italy; 6 Research Department, Division of Clinical Immunology—Infectious Diseases, Fondazione IRCCS Policlinico San Matteo, 27100 Pavia, Italy

**Keywords:** primary myelofibrosis, myeloid-derived suppressor cells, inflammation

## Abstract

**Simple Summary:**

Myeloid-derived suppressor cells (MDSCs) are immature cells that expand in the circulation of patients with cancer, sepsis or chronic inflammation, modulate the immune response against cancer (favoring tumor onset and progression) and promote neoangiogenesis. These premises suggest that MDSCs could be involved in the pathogenesis of primary myelofibrosis (PMF) (a myeloproliferative neoplasm characterized by chronic inflammation and extensive neoangiogenesis in bone marrow and spleen). In this paper, we found that (1) MDSCs are increased both in the circulation and in the spleen of PMF patients and strongly correlate with disease progression; and (2) reduced CXCR4 expression on MDSCs along with increased plasmatic SDF-1α can be involved in their mobilization. These findings suggest that circulating MDSCs can be considered a parameter of disease severity and set MDSCs as potential new targets for cancer therapy.

**Abstract:**

Primary myelofibrosis (PMF) is a myeloproliferative neoplasm characterized by a chronic inflammatory state that plays a relevant role in the disease pathogenesis (as proven by high levels of inflammatory cytokines with prognostic significance and by a persistent oxidative stress) and by extensive neoangiogenesis in bone marrow (BM) and spleen. Myeloid-derived suppressor cells (MDSCs) are immature cells that expand in patients with cancer, sepsis or chronic inflammation, favoring tumor onset and progression mainly through the decrease in immune surveillance and the promotion of neoangiogenesis. In this paper, we evaluated the presence of circulating MDSCs in PMF patients, the plasmatic factors involved in their mobilization/expansion and the correlations with laboratory, genetic and clinical parameters. The data indicated that MDSCs could have a relevant role in PMF as a new pathogenic mechanism contributing to explaining the phenotypic diversity observed during the clinical course of the disease, or a potential new target for personalized treatment.

## 1. Introduction

Primary myelofibrosis (PMF) is a Philadelphia-negative chronic myeloproliferative neoplasm (Ph-MPN) characterized by abnormal proliferation and trafficking of hematopoietic progenitor cells, extramedullary hematopoiesis, variable degrees of bone marrow (BM) fibrosis, splenomegaly, and extensive angiogenesis in BM and spleen [1,2]. An acquired mutation of JAK2, CALR or MPL genes (so-called “driver mutations”) is detectable in hematopoietic cells in >95% of PMF patients. Although JAK-STAT pathway activation due to the driver mutations is considered pivotal in PMF pathogenesis, inflammation is also thought to play a role in its onset and progression as proven by high levels of pro-inflammatory cytokines and by a state of chronic oxidative stress [3,4]. 

Myeloid-derived suppressor cells (MDSCs) are heterogeneous cell populations that expand in the peripheral blood (PB), spleen, liver and lymphoid organs during acute/chronic infections, trauma or sepsis and in tumor microenvironment. Common features of MDSCs are the immature state, the strong ability to reduce cytotoxic functions of T and NK cells and tumor proangiogenic activity [5,6]. In physiologic conditions, immature myeloid cells migrate from the BM to different peripheral organs, where they differentiate into macrophages, dendritic cells or granulocytes [5]. Cytokines and chemokines, produced in the tumor microenvironment, recruit immature myeloid cells in the periphery, prevent their differentiation (through the STAT3 signaling pathway) and induce their activation into MDSCs [5]. The precise mechanisms underlying the mobilization of MDSCs are still under investigation; some papers suggest that the recruitment of MDSCs into the tumor microenvironment is due to CXC chemokines together with different inflammatory cytokines [7]. Although the physiopathology of MDSCs is still partially elusive, there is common agreement that they play a role in tumor pathogenesis, favoring their onset and progression through their immunosuppressive and pro-neoangiogenic activities. In keeping with this notion, previous studies demonstrated that the frequency of circulating MDSCs represents a reliable predictor of a negative outcome and response to therapy both in solid cancers and in hematologic malignancies [7,8]. This is true in particular in solid tumors [9,10,11], whereas in hematologic malignancies, possibly due to their heterogeneity, some issues about their function and mechanism still remain unsolved. In the last years, many experimental attempts to counteract MDSCs’ pro-tumor activity have been pursued by means of drugs that either inhibit their migration from the bone marrow to tumor sites, or favor their reprogramming into non-immunosuppressive cells, or deplete them by chemotherapy (reviewed in [12]).

Recently, an increased frequency of MDSCs was described in the PB of patients with PMF [13,14], though this scenario was not confirmed in the BM of MPN patients where MDSCs have been described as increased [14] or decreased [15] compared to healthy controls. Altogether, these premises suggest that MDSCs, fueled by PMF inflammation background, could be involved in PMF pathogenesis.

In this study, we investigated MDSC subsets (polymorphonuclear (PMN)- and monocytic (M)-MDSCs [16]) in patients with PMF, their relation with clinical characteristics and driver mutations, and the receptors and ligands possibly involved in their mobilization.

## 2. Materials and Methods

### 2.1. Subjects

Forty-one PMF patients and twenty-one healthy subjects (CTRLs) were enrolled in the study at Fondazione IRCCS Policlinico San Matteo. Demographic, hematologic and molecular characteristics of subjects at the time of blood sampling for MDSC detection are detailed in Table 1. Patients with PMF were diagnosed according to the revised 2016 WHO classification [17]. The size of the spleen was measured by ultrasonography and reported as maximum interpolar diameter. DNA of total PB granulocytes was prepared using standard procedures, and the allelic burden of JAK2V617F and CALR mutations was detected by digital droplet PCR (dPCR).

### 2.2. Samples and Flow Cytometry Analysis of MDSCs

PB and platelet-poor plasma samples were obtained from *n* = 41 PMF patients and *n* = 21 CTRLs. 

The 1 × 10^6^ low-density cells obtained after gradient centrifugation (1077 g/mL) were stained within 1 h [18], with BV421-anti-CD11b, PeCy7-anti-CD14, APC-anti-HLA-DR, PerCP-anti-CXCR4 (all Becton Dickinson, Franklin Lakes, NJ, USA), PeCy7-anti-CD15 (Thermo Fisher Scientific, Waltham, MA, USA), PE-anti-LOX1 (BioLegend, San Diego, CA, USA) [19]. Intracellular Arginase-1 (Arg-1) was detected with a specific monoclonal antibody after appropriate fixation and permeabilization steps (Thermo Fisher Scientific). Zero point five-1 × 10^6^ cells were acquired by a flow cytometer (FACSCanto^TM^ II; Becton Dickinson) and analyzed by FACSDiva^TM^ software version 9.0 (Becton Dickinson). MDSC subsets were identified as CD11b^+^CD15^+^Lox1^+^ (PMN-MDSCs) or CD11b^+^HLA-DR^low/−^CD15^−^CD14^+^ (M-MDSCs) (Figure S1) and measured as a percentage of the acquired cells and expressed as median (range).

### 2.3. Detection of Proteins in Plasma

The levels of *n* = 8 cytokines/chemokines (CCL2/MCP-1, CXCL5/ENA-78, FGF-2, IL-1β, IL-6, CXCL8/IL-8, TNF-alpha, VEGF) were measured in undiluted platelet-poor plasma samples by Multiplex Bead Assay Bio-Plex (R&D Systems, Minneapolis, MN, USA) using FLEX MAP 3D (Luminex Corporation, Austin, TX, USA). CXCL12/SDF-1α plasma levels were determined by an enzyme-linked immunosorbent assay (ELISA) (R&D Systems). Plasma levels of soluble urokinase Plasminogen Activator Receptor (suPAR) were estimated using a turbidimetric immunoassay (suPARnostic^®^ kit; ViroGates, Copenhagen, Denmark) on Siemens Advia Xpt (Munich, Germany).

The suPARnostic^®^ TurbiLatex is a quantitative test measuring the suPAR level in ng/mL. 

### 2.4. Histopathology and Immunohistochemistry on Spleen Sections

The analysis of myeloid suppressor cells in the spleen was performed on tissue samples from 22 PMF patients and 20 CTRLs. Patients with PMF underwent splenectomy either because of anemia, symptomatic splenomegaly, or because of both. The spleen specimens were analyzed, reduced, and stored in the histological archive of the Anatomy Pathology Section. Normal spleens samples were obtained from subjects undergoing splenectomy following major traumas and/or spleen rupture (e.g., after a car accident).

Formalin-fixed paraffin-embedded (FFPE) tissue blocks of spleen samples were cut into 3 μm thick slides, and stained with Arginase A1 antibody (Santa Cruz Biotechnologies, Dallas, TX, USA) using the automated platform Dako Omnis Envision Flex according to the manufacturer instructions and the EnVision FLEX, High pH (Agilent, Santa Clara, CA, USA). The percentage of Arginase-1^+^ (Arg-1^+^) cells was estimated on total spleen cellularity and the distribution of positive cells was compared to that of patient and control samples.

### 2.5. Statistical Analysis

Statistical analyses were performed using STATISTICA version 8.0 (StatSoft Inc., Tulsa, OK, USA), and in all calculations, *p* < 0.05 was considered statistically significant.

Continuous variables were checked for normality distribution using the Shapiro–Wilk test. Data did not meet the normality assumption and were thus represented using their median and interquartile range. Categorical variables were represented as absolute and percentage frequencies.

Groups were compared by means of Mann–Whitney U-test for unpaired samples. Correlation coefficients between circulating PMN-MDSCs and clinical/biological parameters were calculated using partial non-parametric correlations (Spearman’s method) that allow for evaluation of the association between two variables after eliminating the effects of age. 

A Receiver Operating Characteristic (ROC) Curve was performed to identify the circulating PMN-MDSCs value with the highest sensitivity and specificity to discriminate between PMF/CTRLs and the Area Under the Curve (AUC) was calculated. The optimal cut-off value was estimated by the Youden Index [20,21]. Additionally, the cut-off was calculated based on the upper reference interval limit estimated as the 97.5th percentile of circulating PMN-MDSCs in the reference population (controls) [22]. 

## 3. Results

### 3.1. Circulating PMN-MDSCs in Patients with PMF and CTRLs

**Figure 1:**
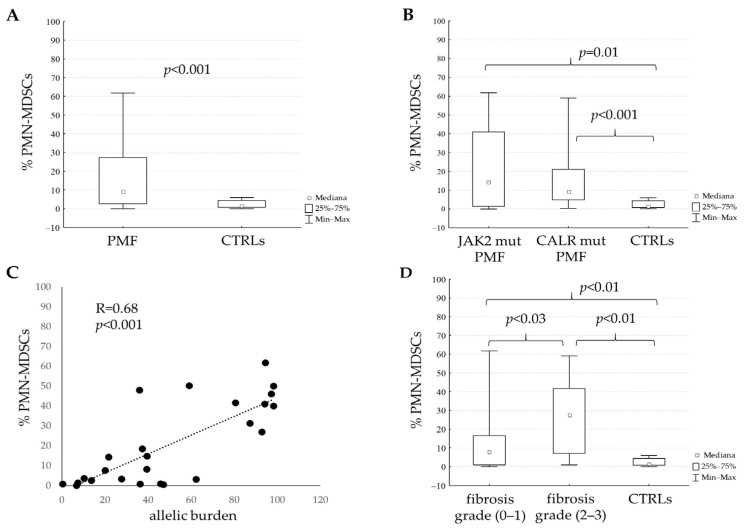
Circulating polymorphonuclear (PMN)-MDSCs in PMF patients and CTRLs. (**A**) PMN-MDSCs, evaluated by flow cytometry, in PMF and CTRLs. (**B**) Analysis of PMN-MDSCs in PMF, according to the mutational status, and CTRLs. (**C**) Correlation between the allelic burden in JAK2-mutated PMF and the percentage of PMN-MDSCs. (**D**) Frequency of PMN-MDSCs according to the BM fibrosis grade in PMF.

In PB mononuclear cells, the percentage of PMN-MDSCs (identified as CD11b^+^CD15^+^Lox1^+^) was higher (*p* < 0.001) in PMF patients (*n* = 41; mean 18% ± 18.7 SD) than in CTRLs (*n* = 21; mean 2.3% ± 2 SD) (Figure 1A); this subset was also characterized by the intracellular expression of Arg-1, comparable in PMF (median 91%; range 80–98) and CTRLs (median 97%; range 94–100). On the contrary, the percentage of circulating M-MDSCs (identified as CD11b^+^HLA-DR^low/−^CD15^−^CD14^+^) was not significantly different between PMF (median 0.4%; range 0.02–3.8) and CTRLs (median 0.4%; range 0.07–1.5). Thus, PMN-MDSCs only are significantly increased in PMF patients compared to CTRLs, whereas the M-MDSC subset is similar to CTRLs and hardly detectable. Since some patients were treated with JAK-inhibitors, we checked the influence of this drug on the circulating levels of PMN-MDSCs; the data showed that the frequency was not significantly different, although increased, in PMF patients under therapy with JAK-inhibitors (median 40%; range 0.8–50) and in untreated or hydroxycarbamide-treated patients. 

We then evaluated circulating PMN-MDSCs according to the mutational status and we found that both JAK2V617F and CALR-mutated patients had higher levels of PMN-MDSCs (*p* = 0.01 and *p* < 0.001, respectively) than CTRLs (Figure 1B). Furthermore, JAK2V617F-homozygous patients had higher levels of PMN-MDSCs (*n* = 11; median 41%; range 3.1–62) with respect to JAK2V617F-heterozygous patients (*n* = 16; median 2.9%; range 0.03–48), CALR-mutated patients and CTRLs (*p* < 0.01 for all). 

We also found that, in JAK2-mutated patients, circulating PMN-MDSCs had a direct correlation with the allelic burden (Figure 1C), whereas no such correlation was found in CALR-mutated patients probably due to the low range of the allelic burden. 

PMF patients with BM fibrosis grade 0–1 had circulating PMN-MDSCs lower (*p* < 0.03) than patients with fibrosis grade 2–3, and both groups had significantly higher (*p* < 0.01) PMN-MDSCs than CTRLs (Table 1 and Figure 1D); moreover, the fibrosis grade directly correlated (R = 0.34; *p* = 0.03) with the percentage of circulating PMN-MDSCs. 

### 3.2. Plasma Levels of Proteins and Analysis of CXCR4 Receptor on PMN-MDSCs

In plasma samples obtained from PMF patients (*n* = 35) and CTRLs (*n* = 9) previously analyzed for the detection of circulating PMN-MDSCs, we evaluated the levels of some of the cytokines and chemokines [7,12] involved in the process of MDSC differentiation, maturation or function. FGF2, IL-6, TNF-α, VEGF and SDF-1α were significantly higher in patients than in CTRLs, while we found comparable levels of CCL2, CXCL5, IL-1β and IL-8 (Table 2). These cytokine significances are therefore to be considered an indirect indicator of MDSC involvement in PMF.

**Figure 2:**
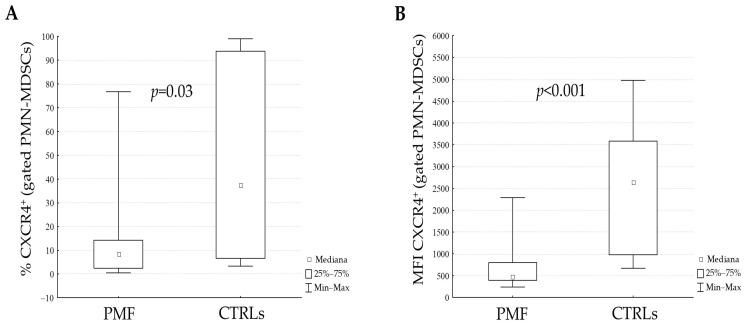
Expression of CXCR4 on circulating PMN-MDSCs. The expression of CXCR4, evaluated as percentage (**A**) and mean fluorescence intensity (MFI) (**B**), on electronically gated PMNMDSCs in PMF patients and CTRLs.

In PMF patients there was a direct statistically significant correlation between SDF-1α and PMN-MDSCs (R = 0.34; *p* < 0.04) and the correlation was even stronger in JAK2-mutated patients (R = 0.45; *p* = 0.024). In light of the well-known role of CXCR4 in PMF [22], we investigated the presence of this receptor on PMN-MDSCs and we found that PMF patients had lower CXCR4 than CTRLs both as a percentage (Figure 2A) and as mean fluorescence intensity (MFI) (*p* = 0.03 and *p* < 0.001, respectively) (Figure 2B). PMN-MDSCs directly correlated with the percentage of CXCR4 (R = 0.46; *p* = 0.01) and inversely with the MFI (R = −0.41; *p* = 0.03); however, there was no correlation between SDF-1α and the expression of CXCR4 on PMN-MDSCs, both as a percentage and MFI. 

Usually, CRP is used as a marker for acute and chronic inflammation, especially in the clinical setting. However, suPAR has been recently reported as a promising biomarker for systemic chronic inflammation, since it is a protein minimally affected by acute changes and short-term influences, in contrast to many currently used markers of systemic inflammation (e.g., CRP, IL-6, and TNF-α) [23]. For this reason, we used suPAR and, interestingly, we found that PMF patients had significantly higher (*p* = 0.01) levels of suPAR (median 4.3 ng/mL; range 2.7–8.8) than CTRLs (median 3.1 ng/mL; range 2.1–4.4). 

### 3.3. Correlations between Circulating PMN-MDSCs and Clinical/Biological Parameters in PMF Patients and Determination of the Cut-Off Value

At variance with previous papers [13,14], we found that circulating PMN-MDSCs correlated directly with disease duration, circulating CD34^+^ cells (percentage and absolute number), white blood cell count, LDH, and spleen size and inversely with the hemoglobin level, the platelet count, and the expression of CXCR4 on circulating CD34^+^ cells (Table S1). Considering that in PMF, the percentage of PMN-MDSCs was directly correlated with the age of patients (R = 0.61; *p* < 0.001), we analyzed the data using partial non-parametric correlations after eliminating the effects due to the age: the analysis confirmed the correlations of the parameters listed above, except for the one between PMN-MDSCs and the spleen size (Table 3).

**Figure 3:**
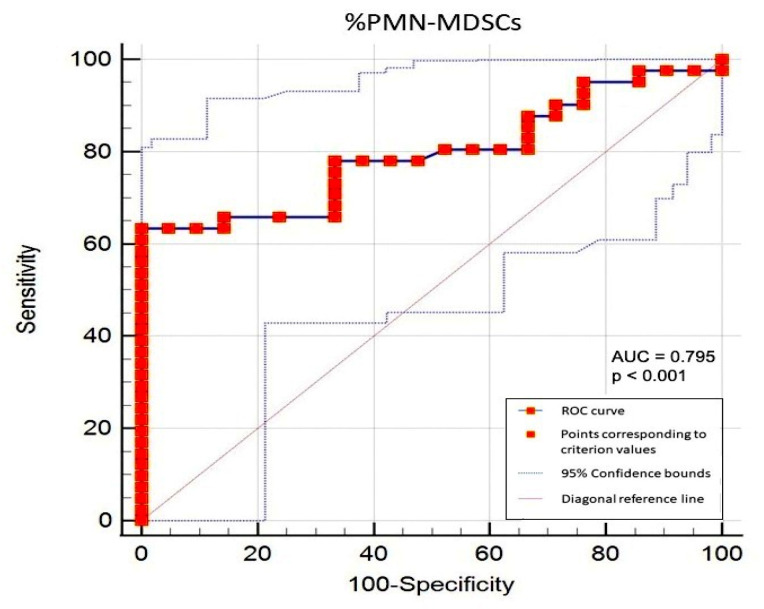
Receiver Operating Characteristic (ROC) Curve. Youden index calculated as: sensitivity + specificity – 1. AUC = Area Under the Curve.

Finally, employing the Youden index, we determined a cut-off of 6% in circulating PMN-MDSCs able to discriminate PMF and CTRLs (Figure 3), and this result was confirmed also by applying a different statistical method [22] (see Section 2 and Table S2). Circulating PMN-MDSCs higher than 6% identified patients with a specificity of 100% (95%, C.I.: 83.9–100) and a sensitivity of 63.4% (95%, C.I.: 46.9–77.9).

### 3.4. Myeloid Suppressor Cells in the Spleen of PMF Patients

Although the correlation between circulating PMN-MDSCs and spleen size is lost after eliminating the age effect, we performed a topographical analysis of the spleen due to its role in the immunity and in the disease progression of PMF. In addition, previous papers showed that MDSCs have the potential to differentiate into endothelial cells and incorporate in tumor endothelium in mice, favoring neoplastic neoangiogenesis [24,25]. 

Even though they were not the same subjects evaluated for the detection of circulating PMN-MDSCs, we took advantage of spleen slides stored at our hospital and targeted Arg-1^+^ myeloid cells [15,26] in spleen sections obtained from PMF patients (*n* = 22) and healthy subjects (*n* = 20). We observed a higher (*p* < 0.001) percentage of Arg-1^+^ cells, calculated on total spleen cells, in PMF (median 22.5%, range 5–60) than in CTRLs (median 7%, range 2–20); moreover, Arg-1^+^ cells directly correlated with the spleen size (R = 0.57, *p* = 0.01) in PMF patients. In addition, although not statistically different from each other, both JAK2-mutated (*n* = 18; median 30%, range 5–60) and CALR-mutated patients (*n* = 4; median 17.5%, range 10–20) had higher Arg-1^+^ cells in spleen sections (*p* < 0.01, for both groups) than CTRLs. 

**Figure 4:**
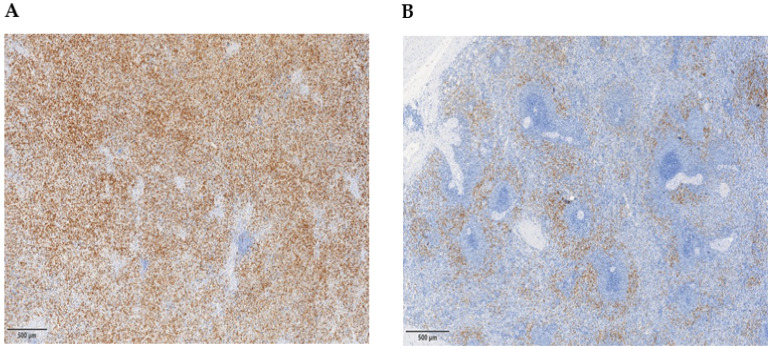
Arginase-1 (Arg-1) immunohistochemical staining of spleen sections. ((**A**), 50× magnification) In PMF patients the spleen architecture was disrupted by the presence of an abundant population of immature myeloid cells frequently expressing Arg-1. ((**B**), 50× magnification) In healthy controls, the follicular structures of the white pulp are preserved and a minor population of Arg-1^+^ cells are observed only at the periphery of spleen follicles.

In PMF, the topographic analysis of the spleen showed an altered architecture due to the infiltration of trilinear hematopoietic cells, and an atrophic structure of the white pulp in the majority of patients. Compared to normal controls, in the spleen samples obtained from PMF, Arg-1 stained a higher number of cells that morphologically resemble band cells and monocytes and were haphazardly distributed all over the red pulp and intermingled with the hematopoietic cells (Figure 4A). Arg-1 staining in normal spleen highlighted a population of cells tidily surrounding the white pulp periarteriolar lymphoid sheaths in the marginal zone. Morphologically, the stained cells were represented by band cells, neutrophils and a few mononucleated cells resembling monocytes (Figure 4B).

## 4. Discussion

In the present study, we showed that in PMF patients, the percentage of circulating MDSCs was higher than in healthy subjects and correlated with disease severity. In contrast with previous reports [8], we first demonstrated that only the PMN-MDSC subset is significantly increased and consistent in PMF patients compared to the controls, whereas the M-MDSC subset is similar to the controls and hardly detectable. Although PMN-MDSCs and neutrophils share common characteristics (e.g., arginase-mediated arginine depletion) [16,27], various functional differences between steady-state neutrophils and PMN-MDSCs were found, including a higher activity of arginase, myeloperoxidase and reactive oxygen species, a reduced expression of CD16 and CD62L, and fewer granules in PMN-MDSCs compared to neutrophils [16,28]. Moreover, this MDSC subset has been shown to not only elicit immunosuppressive mechanisms comparable to M-MDSCs, but also to possess unique tumor and metastasis-promoting properties, as shown in previous works on solid tumors [12,29,30,31,32,33,34]. Considering the negative prognostic value that increased levels of PMN-MDSCs specifically have in many tumors, such as for instance melanomas, colorectal cancers, and non-small cell lung cancers [30,31,34], the selective increase in this subset of MDSCs in PMF patients, besides playing a role in the chronic inflammatory status that characterizes the disease and/or in the migration of malignant CD34^+^ cells from the PB toward the spleen, could also be relevant for a prognostic stratification of the patients. Our observation that the frequency of circulating PMN-MDSCs increases with the worsening of the disease speaks in favor of this hypothesis.

In our cohort, the correlation with the allelic burden in JAK2-mutated patients indicates a link between the size of the clone and the number of circulating PMN-MDSCs, suggesting a role for the mutational status in determining the expansion of this population. Previous studies on circulating MDSCs in MPN patients never found a correlation with genotype, which was likely due to the inclusion in their cohort of patients with both primary and secondary myelofibrosis, as well as polycythemia vera and essential thrombocythemia, together with the assessment of MDSCs as a whole population and not as separate subsets [13,14]. However, in the BM of MPN patients, a reduced frequency of MDSCs, assessed by Arginase-1 expression only, has been correlated with the presence of a CALR genotype [15]. In contrast, Kapor et al. found, in the BM of MPN patients, an increased frequency of MDSCs (identified as CD33^+^HLA-DR^low/−^CD15^+^CD14^−^) and no correlation with genotype [14]. These discrepancies could be due to their different methodological approach as well as to the different composition of the cohort of patients.

Although some patients can remain in a pre-fibrotic phase for several years, PMF is usually characterized by the progressive deposition of an extracellular matrix, produced by stromal cells, resulting in an uncontrolled stromal cell activation leading to BM fibrosis, which interferes with the residual hematopoiesis and gradually worsens the disease outcome [35]. Our data showed a direct correlation between circulating PMN-MDSCs and the fibrosis grade, suggesting the contribution of these cells in the disease worsening. Alternatively, fibrosis itself could be the cause of the leakage of immature myeloid cells from the BM.

Previous papers have identified CXCR4 as an important mediator of MDSC recruitment, regulated by an autocrine production of cytokines [36,37], and its ligand, SDF-1α, leads to the activation of the downstream AKT pathway and mediates the reduction in MDSC apoptosis [38]. Surprisingly, the lower expression of CXCR4 on PMN-MDSCs in PMF than in CTRLs is in contrast with what was observed in solid tumors, where CXCR4 is frequently increased [12,39,40]. As for circulating CD34^+^ cells [22], CXCR4^+^ PMN-MDSCs were less than in CTRLs, suggesting that a general decrease of this receptor in PMF could be a biological feature of the disease, deserving further investigations. An intriguing hypothesis is that the low expression of CXCR4 on PMN-MDSCs together with the increased levels of plasmatic SDF-1α could be part of the mechanism responsible for the mobilization of PMN-MDSCs from the BM to the PB.

PMN-MDSCs are strongly associated with disease status, their frequency increasing with the progression of the disease, independently from treatment and age of subjects. This suggests that the frequency of circulating PMN-MDSCs can be considered a parameter of disease severity as it occurs in patients with myelodysplastic syndromes, where the frequency of circulating MDSCs increases from low-risk patients toward high- and very high-risk patients [41]. At variance from what is observed in most solid tumors and some hematologic malignancies, where M-MDSCs are always detectable at levels similar or sometimes higher than PMN-MDSCs [41]; in PMF, this subset was hardly detectable. Moreover, we determined a cut-off value of 6% in circulating PMN-MDSCs that discriminates PMF and CTRLs.

According to previous publications, it is well-known that PMN-MDSCs rely to a large degree on the expression of Arg-1 to exert an immunosuppressive capacity [42,43] and that angiogenic factors in the tumor microenvironment can induce the differentiation of myeloid immune suppressor cells toward endothelial cells [24]. In the spleen tissue obtained from patients, the large number of myeloid suppressor cells and their correlation with the size of the organ pointed out a possible role of these cells in the spleen neoangiogenesis that characterizes the disease. In addition, the characteristic myeloid suppressor cell distribution in spleen tissue could be either related to the altered architecture of the organ due to splenomegaly or be a consequence of the migration to the spleen of myeloid cells from the PB.

## 5. Conclusions

MDSCs have been a matter of increasing investigation in the last years, being considered major players in the inflammatory milieu that characterizes the onset and progression of cancer. In this work, we have expanded on previous observations that circulating MDSCs are increased in PMF patients, showing that this increase is due to PMN- rather than to the M-MDSCs. We have also added new evidence with regard to correlation with genotype, clinical and biological parameters of the disease and we have proposed a potential mechanism of mobilization. On the other hand, we did not perform functional assays confirming the immunosuppressive activity of MDSCs in PMF. Thus, our data do not allow a definitive explanation of the role of MDSCs in PMF onset and progression and further investigations are needed to clarify such a role. If the involvement of MDSCs in PMF pathogenesis can be defined, then MDSCs could become a target for therapeutic strategies aimed at favoring their differentiation into mature PMN cells, or at counteracting their immunosuppressive and neoangiogenic activity, or at inhibiting their ability to migrate from the BM to peripheral organs, in particular, the spleen. 

## Figures and Tables

**Table 1:** The upper part of the table illustrates demographic and therapy-related data of patients with primary myelofibrosis (PMF) and controls (CTRLs) at the time of blood sampling for myeloid-derived suppressor cell (MDSC) detection. The other sections of the table illustrate the clinical–hematological, biological and molecular characteristics of PMF patients.

	Primary Myelofibrosis (PMF) (PMF)	Controls (CTRLs)
Number of subjects	41	21
Age (years), median (range)	54 (36–83)	58 (28–90)
Out of therapy, number (percent)	15 (36.5%)	21 (100%)
In therapy, number		
- hydroxycarbamide	17	0
- JAK-inhibitors	9	0
- statin only	1	0
- hydroxycarbamide + statin	1	0
- prednisolone	1	0
Survival *, number (percent)	37 (90.2%)	na
**PMF clinical–hematological characteristics**		
Hemoglobin (g/L), median (range)	124 (80–157)	
White-blood cell count (×10^9^/L), median (range)	8 (2–39.6)	
Platelet count (×10^9^/L), median (range)	430 (38–1400)	
LDH ^#^ (mU/mL), median (range)	299 (127–1322)	
Blasts ^@^, number of PMF (percent)		
- 0	32 (91.5%)	
- 1	1 (2.8%)	
- ≥2	2 (5.7%)	
CD34^+^ absolute number/µL, median (range)	7.6 (0.6–2855)	
**PMF biological and molecular characteristics**		
Bone marrow (BM) fibrosis, number (percent)		
- grade 0–1	24 (58.5%)	
- grade 2–3	17 (41.5%)	
Spleen size (cm), median (range)	120 (90–437)	
JAK2V617F mutation, number (percent)	27 (65.8%)	
CALR mutation, number (percent)	14 (34.2%)	

na = not available; * last follow-up December 2023; ^#^ LDH available in 29 PMF; ^@^ number of blasts available in 35 PMF.

**Table 2:** Plasmatic levels of cytokines and chemokines in PMF patients and CTRLs. Data, expressed in pg/mL, are shown as median (range). NS = not significant.

	PMF	CTRLs	*p*
CCL2	123.6 (17.6–282)	159 (73.9–212)	NS
CXCL5	163 (15.4–1073)	173 (37.4–1076	NS
FGF2	4.2 (<0.5 to 10.9)	<0.5 (<0.5 to 9.2)	0.002
IL-1β	<5.9 (<5.9 to 35.1)	<5.9 (<0.5 to 9.3)	NS
IL-6	1.3 (<0.4 to 29.3)	<0.4 (<0.4 to 1.3)	<0.001
IL-8	4.5 (<1.3 to 34.4)	1.9 (<1.3 to 6.7)	NS
TNF-α	6.5 (<2.4 to 31.5)	<2.4 (<2.4 to 3.4)	<0.001
VEGF	26.2 (3.9–140)	5.8 (4.3–22.3)	<0.001
SDF-1α	2155 (1313–3325)	1730 (1145–2606)	0.02

**Table 3:** PMN-MDSCs and clinical parameters. Correlation coefficients, between circulating PMN-MDSCs and clinical/biological parameters, using partial non-parametric correlations (Spearman’s method) to evaluate the association between two variables after eliminating the effects due to the association with a third variable (age).

	*n*	R	*p*
% CD34^+^	41	0.403	0.01
CD34 absolute number/µL	38	0.566	<0.001
WBC count (×10^9^/L)	39	0.410	0.01
LDH (mU/mL)	29	0.463	0.01
Hb level (g/L)	39	−0.419	<0.01
Plt count (×10^9^/L)	39	−0.353	0.03
% CXCR4^+^ (on gated CD34^+^)	41	−0.485	<0.01

WBC = white blood cells; LDH = lactate dehydrogenase; Hb = hemoglobin; Plt = platelets.

## Data Availability

The data presented in this study are available on request from the corresponding author.

## Supplementary Materials

The following supporting information can be downloaded at: https://www.mdpi.com/article/10.3390/cancers16142556/s1, Figure S1: Gating strategy to identify PMN- and M-MDSCs; Table S1: Correlations between circulating PMN-MDSCs vs. clinical/biological parameters; Table S2: Categorization according to a cut-off value of 6%.
