# Supplementary material for: Circulating Polymorphonuclear Myeloid-Derived Suppressor Cells (PMN-MDSCs) Have a Biological Role in Patients with Primary Myelofibrosis
Source: Cancers (Basel). 2024 Jul 16;16(14):2556. doi: 10.3390/cancers16142556 (PMC11275082; doi:10.3390/cancers16142556)
Supplement: Supplementary file 1 [file cancers-16-02556-s001.zip › cancers-3039247-supplementary.pdf]

Supplementary Material

Figure S1. Gating strategy to evaluate PMN-MDSCs (CD11b$^+$CD15$^+$Lox1$^+$) and M-MDSCs (CD11b$^+$HLA-DR$^{low/-}$CD15$^-$CD14$^+$) in peripheral blood of PMF patients and CTRLs by flow cytometry.

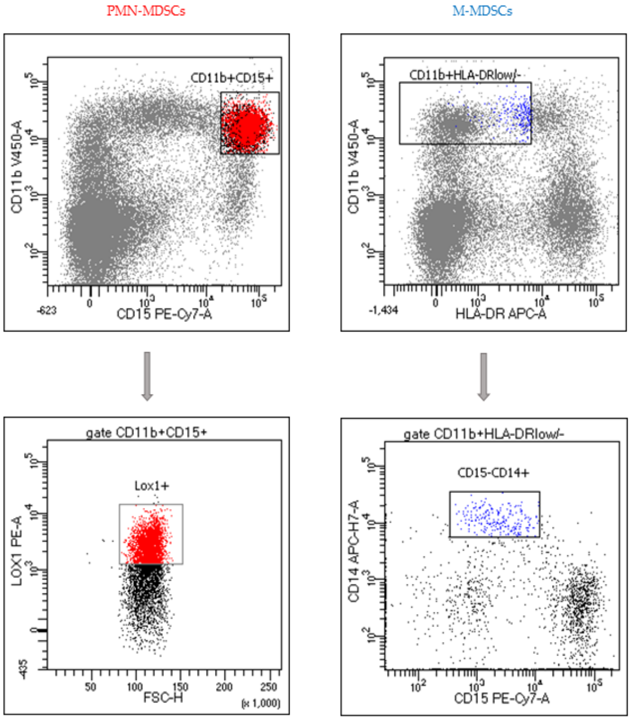

Table S1. Correlations between the percentage of circulating PMN-MDSCs and clinical/biological parameters in PMF patients.

	n	R	p
Disease duration (months)	41	0.32	0.04
% $CD34^{+}$	41	0.49	0.001
CD34 absolute number/μl	38	0.60	<0.001
WBC count ($x10^{9}$/L)	39	0.42	0.008
LDH (mU/ml)	29	0.55	<0.01
Spleen size (cm)	40	0.44	<0.01
Hb level (g/L)	39	-0.52	<0.001
Plt count ($x10^{9}$/L)	39	-0.49	<0.001
% $CXCR4^{+}$ (on gated $CD34^{+}$)	41	-0.58	<0.001

WBC = white blood cells
Hb = hemoglobin
Plt = platelets

Table S2. Categorization according to a cut-off value of 6%. True condition against PMF and CTRLs categorization according to a cut-off value of 6% based on the higher reference interval limit estimated as the 97.5th percentile distribution of circulating PMN-MDSCs in healthy subjects.

		Categorization according to a cut off value of 6%		
		PMF	CTRLs	Total
True condition	PMF	26 (100.0%)	15 (41.7%)	41 (66.1%)
	CTRLs	0 (0.0%)	21 (58.3%)	21 (33.9%)
	Total	26 (100.0%)	36 (100.0%)	62 (100.0%)
